# Is HBV viral load at admission associated with development of acute-on-chronic liver failure in patients with acute decompensation of chronic hepatitis B related cirrhosis?

**DOI:** 10.1186/s12879-019-3988-1

**Published:** 2019-04-30

**Authors:** Jian-Hua Lei, Feng Peng, Zi Chen, Xin-Qiang Xiao

**Affiliations:** Department of Infectious Diseases, the Second Xiangya Hospital, Central South University, No.139 Middle Renmin Road, Changsha, Hunan 410011 People’s Republic of China

**Keywords:** Hepatitis B, chronic, Acute-on-chronic liver failure, Viral load, Nucleoside/nucleotide analogue

## Abstract

**Background:**

Hepatitis B virus (HBV) reactivation is one of the most common precipitating events associated with acute decompensation (AD) or acute-on-chronic liver failure (ACLF) in chronic hepatitis B (CHB)-related cirrhotic patients. However, whether their serum HBV deoxyribonucleic acid (DNA) levels are associated with ACLF incidence and short-term mortality rate is still ambiguous.

**Methods:**

The ACLF incidences, 28-day and 90-day liver transplantation (LT)-free mortality rates, previous nucleoside/nucleotide analogues (NUCs) treatments and serum HBV DNA levels at admission (ad-levels) of 111 hospitalized patients with AD of CHB-related cirrhosis were analyzed.

**Results:**

43 (38.7%) patients developed ACLF. The 28-day and 90-day LT-free mortality rates of the ACLF cases were 15.4 and 40.9%, respectively. Though NUCs inhibited HBV replication effectively, there were no differences in the ACLF incidence between antiviral treatment-naïve patients and NUCs treatment-experienced patients with or without interruptions (37.5, 41.7 and 45.5%, respectively, *P*>0.05). The serum HBV DNA ad-level was similar between the patients with and without ACLF development (logarithms: 4.50 ± 1.96 vs 4.32 ± 1.99; ≥2000 IU/ml: 67.4% vs 67.6%; both *P*>0.05), so was between the ACLF patients died or survived in 28 or 90 days (logarithms: 4.31 ± 1.91 vs 5.54 ± 2.53, 4.81 ± 1.76 vs 4.84 ± 2.40, respectively, both *P*>0.05).

**Conclusion:**

Serum HBV DNA ad-level and previous NUCs treatment are not associated with incidence of ACLF and short-term mortality rate in the hospitalized patients with AD of CHB-related cirrhosis.

## Background

Some compensated or decompensated cirrhotic patients might develop to a stage of acute-on-chronic liver failure (ACLF) characterized by hepatic and/or extrahepatic organ failures and high short-term mortality [1, 2]. In China, ACLF frequently occurs in patients with exacerbation of acute decompensation (AD) of chronic hepatitis B (CHB)-related cirrhosis [3–5].

Strong associations were found between hepatitis B virus (HBV) deoxyribonucleic acid (DNA) level and development of cirrhosis and hepatocellular carcinoma (HCC) in chronic HBV infected patients [6–8]. Control of HBV replication by nucleoside/nucleotide analogues (NUCs) therapies could reduce the risk of HCC and decompensated liver disease among CHB-related cirrhotic patients [9, 10]. However, whether HBV DNA level is associated with incidence of ACLF and short-term mortality rate in acutely decompensated CHB-related cirrhotic patients is ambiguous [3, 4, 11].

As inhibiting replication of HBV in CHB-related cirrhotic patients by NUCs treatment is recommended by most authoritative guidelines [12–15], analysis on associations between NUCs treatment program, serum HBV DNA level, ACLF incidence and short-term mortality rate among hospitalized CHB-related cirrhotic patients would be helpful to understand the role of NUCs therapies in treatment schemes and to formulate schemes to prevent the occurrence of ACLF.

## Methods

### Patients and ethics approval

111 patients with AD of CHB-related cirrhosis hospitalized in the infectious disease wards of the Second Xiangya Hospital, Central South University, during July and December 2016 were enrolled in the study. Data were obtained from the written medical records. The incidences of ACLF, 28-day and 90-day liver transplantation (LT)-free mortality rates, previous NUCs treatments and serum HBV DNA levels at admission (HBV DNA ad-levels) of the patients were analyzed.

The collection of the demographic data, clinical features and biological samples including serum, plasma and LT-resected liver tissue samples of the patients fulfilled the requirements of medical ethics. The ethical review committee of the hospital approved this study. Written informed consent was obtained from all participants.

### Diagnoses of CHB, liver cirrhosis and AD events

CHB was diagnosed according to the guidelines [12–15]. Patients with evidence of HCC or other chronic liver disease were excluded.

Liver cirrhosis was diagnosed by histological examination of the LT-resected liver, or FibroScan liver stiffness detected within 6 months more than 14.1 kilopascal together with Child-Turcotte-Pugh (CTP) scores more than 7 at admission, or imaging signs of nodular liver together with any two of the following criteria: the presence of ascites, hepatic encephalopathy, upper gastrointestinal bleeding, endoscopic detection of gastroesophageal varices, splenomegaly and peripheral blood platelet count below 100 × 10^9^/L in the absence of other explanations [15–19].

AD events such as acute exacerbation of hepatic damage, overt ascites, upper gastrointestinal bleeding, hepatorenal syndrome and bacterial infection were defined according the clinical practice guidelines [20–22]. A model for end-stage liver disease (MELD) score assessing the severity of chronic liver injury was calculated in every patient on the first day of hospitalization according to the Malinchoc formula in which the levels of serum bilirubin, creatinine and international normalized ratio were analyzed simultaneously [17, 23].

### Diagnosis and grades of ACLF

Diagnostic criteria and grades of ACLF were defined according to EASL-CLIF Consortium definition [1]. According to the consensus recommendations on ACLF of the Asian Pacific Association for the Study of the Liver (APASL) and studies of the domestic scholars [2–5, 24–26], the current study defined the observation period of the incidence of ACLF development as 4 weeks following the onset of the AD events.

To evaluate the occurrence of organ failure or dysfunction in the patients, a retrospective analysis was performed every day in every patient by use of the CLIF-SOFA scale [1, 27]. According to the scale, liver failure was defined by a serum bilirubin level of ≥204 μmol/L, renal failure was defined by a serum creatinine level of ≥176 μmol/L or the need for renal replacement therapy, renal dysfunction was defined by a serum creatinine level between 132 μmol/L and 176 μmol/L, cerebral failure was defined by grade III-IV hepatic encephalopathy, coagulation failure was defined by a INR ≥2.5 or a platelet count of ≤20 × 10^9^/L, respiratory failure was defined by a ratio of partial pressure of arterial oxygen (PaO2) to fraction of inspired oxygen (FiO2) of ≤200 or an pulse oximetric saturation (SpO2) to FiO2 ratio of ≤214, circulatory failure was defined by a mean arterial pressure<70 mmHg despite adequate fluid resuscitation and vasoconstrictor requirements. Patients who had single kidney failure or single non-renal organ failure with presence of kidney dysfunction and/or grade I-II hepatic encephalopathy were diagnosed with ACLF grade 1. Patients who had two or more than two organ failures were diagnosed with ACLF grade 2 and ACLF grade 3, respectively.

### Quantitatively detection of serum HBV DNA

Quantitation of serum HBV DNA was assessed in StepOnePlus real-time polymerase chain reaction (PCR) system (Applied Biosystems Inc., USA), by using HBV DNA quantitative fluorescence diagnostic kit (Sansure biotech Inc. Ltd., China) [28]. The lower limit of the detection was 10 IU/mL and the linear range was from 20 IU/mL to 2.0 × 10^9^ IU/mL.

### Statistical analysis

All statistical analyses were performed with IBM® SPSS® Statistics version 20.0, using descriptive statistical indexes such as mean, standard deviation (SD), range, median, mortality rate, constituent ratio, et al. Chi-squared test, Fisher’s exact test, Analysis of Variance, LSD multiple comparisons tests and Kruskal-Wallis H test were performed for comparison of means or ratios. The homogeneity test of variance of the quantitative data was performed by Levene test. Binary logistic analyses of the 28-or 90-day mortality rates in patients without LT treatment were performed. The *P* values in this paper were all derived from the double tailed tests and P value less than 0.05 was considered statistically significant.

## Results

### Prevalence of ACLF and the 28-day and 90-day mortality rates

According to the EASL-CLIF diagnostic criteria, 3 patients were diagnosed with ACLF grade 1, 27 with ACLF grade 2 and 13 with ACLF grade 3. The prevalence rate of ACLF was 38.7%. 26 patients were diagnosed with ACLF at admission and 17 patients developed ACLF during hospitalization. The prevalence rates of liver failure (54.9%) and coagulation failure (32.4%) were significantly higher than the rates of cerebral failure (11.7%), renal failure (7.2%), circulatory failure (6.3%) and respiratory failure (3.6%) (*P*<0.05). The prevalence rates of renal and cerebral dysfunction were 5.4 and 3.6%, respectively. Liver failure was observed in 21 (30.9%) of the 68 patients without ACLF development while extrahepatic organ failure was rarely found.

The 28-day and 90-day LT-free mortality rates of the patients without ACLF development were both zero. One patient without extrahepatic organ failure developed liver failure and received LT treatment on the 55th day of hospitalization and recovered.

Detailed 28-day medical records were obtained in 42 of the 43 ACLF cases. 3 (7.1%) underwent LT within 28 days and survived till the 90th day. 6 (15.4%) of the rest 39 patients without LT treatment died before the 28th day. 8 (26.7%) of the 30 cases with detailed 90-days medical records were treated with LT, one (12.5%) of them died before the 90th day. 9 (40.9%) of the rest 22 patients without LT treatment died before the 90th day.

None of the 3 cases diagnosed with ACLF grade 1 was treated with LT, one patient died within 28 days and another died during the 28th and the 90th day. The 28-day or 90-day LT-free mortality rates of the cases diagnosed with ACLF grade 2 were zero and 21.4%, respectively. The 28-day or 90-day LT-free mortality rates of the cases diagnosed with ACLF grade 3 were 41.7 and 83.3%, respectively.

### Gender and age distribution, MELD scores at admission

Gender and age distributions were not significantly different in the patients with ACLF or without (constituent ratio of males: 90.7% vs 77.9%; age: 47.4 ± 11.2 vs 47.6 ± 11.4 years. Both *P*>0.05). MELD scores at admission were significantly higher in the patients who developed ACLF than those did not (26.6 ± 5.4 vs 14.2 ± 7.4, *P*<0.05). Among the 85 patients who did not develop ACLF before admission, 17 patients developing ACLF during hospitalization had higher MELD scores (22.4 ± 3.0 vs 14.2 ± 7.4, *P*<0.05). MELD scores of the patients who developed ACLF after admission were lower than those who were diagnosed with ACLF at admission (22.4 ± 3.0 vs 29.3 ± 4.9, *P*<0.05).

### PEs associated with AD or ACLF

Potential PEs associated with AD or ACLF were present in 56 cases (50.5%). The most frequent PE was bacterial infection (19 cases, 17.1%), followed by overwork (12 cases, 10.8%), interruption/discontinuation of antiviral treatment (10 cases, 9%), suspected drug-induced liver injury (6 cases, 5.4%) and excessive alcohol consumption (5 cases, 4.5%). 9 cases (8.1%) had infrequent PEs like flu or cold (3 cases), acute HEV infection (2 cases), resistance to NUCs (2 cases), complicated hyperthyroidism (1 case) and use of immunosuppressive agents (1 case).

The ACLF incidence was not significantly different between the patients with or without presence of PE (41.9% vs 34.7%, *P*>0.05). 7 of the 13 patients who had PEs of NUCs resistance or interruption/discontinuation of antiviral treatment developed ACLF, but their ACLF incidence was not statistically different from the others’ (53.9% vs 36.7%, *P*>0.05). However, only 2 of the 12 patients with overwork as PE developed ACLF, showing a lower ACLF incidence than the others (16.7% vs 48.0%, Fisher’s exact test, *P* = 0.05).

### Incidence of ACLF in patients with different previous NUCs treatment experiences (Table 1)

88 cases (79.3%) were antiviral treatment-naïve, NUCs therapies were started in them when the symptoms of AD were obvious.87 of them were treated with entecavir (ETV) as soon as they were admitted to the hospital. One patient failed to receive NUCs treatment due to intestinal perforation and peritonitis.

**Table 1:** Incidence of ACLF in patients with different previous NUCs treatment experiences

NUCs experiences	HBV DNA ad-level (IU/ml), n (%)		Log (HBV DNA), mean ± SD	MELD scores, mean ± SD	ACLF development, n (%)			
	<2000	≥2000			n	before admission	after admission	Total
naïve	26 (29.5)	62 (70.5)	4.55 ± 1.71	19.7 ± 8.9	88	22 (25.0)	11 (12.5)	33 (37.5)
maintained	8 (66.7)	4 (33.3)	2.27 ± 2.48	17.5 ± 9.9	12	2 (16.7)	3 (25.0)	5 (41.7)
interrupted	2 (18.2)	9 (81.8)	5.40 ± 1.90	15.4 ± 8.8	11	2 (18.2)	3 (27.3)	5 (45.5)
Total	36 (32.4)	75 (67.6)	4.39 ± 1.97	19.0 ± 9.0	111	26 (23.4)	17 (15.3)	43 (38.7)

1. NUCs experiences: naïve: antiviral treatment-naïve patients; maintained: patients with sustained long-term NUCs treatments till admission; interrupted: patients with interruption or discontinuation of antiviral treatment after long-term of NUCs therapies

2. HBV DNA ad-level <2000 IU/ml or ≥ 2000 IU/ml: Fisher’s exact test, *P* = 0.032

3. Log(HBV DNA) (logarithm of the serum HBV DNA ad-level): F = 10.150, *P* = 0.000

4. MELD scores (MELD scores at admission): F = 1.301, *P* = 0.276

5. ACLF development: Fisher’s exact test, *P* = 0.549; ACLF vs No-ACLF: χ^2^ = 0.309, *P* = 0.828; ACLF after admission vs No-ACLF: Fisher’s exact test, χ^2^ = 2.083, *P* = 0.353; ACLF before admission vs No-ACLF: Fisher’s exact test, *P* = 1.000

Twelve patients (10.8%) were previously treated with NUCs for long-terms (range from 14 to 286 weeks, median: 58 weeks) till their admissions to the hospital, without interruptions. The NUCs treatment schemes were persisted during their hospitalizations. 8 of them were treated with ETV, 2 with lamivudine (LAM), consistently. One patient was previously treated with ETV for 75 weeks and then sequentially treated with ETV and tenofovir disoproxil fumarate (TDF) due to a confirmed ETV resistance mutation. Another patient was previously treated with LAM for 1 year and then sequentially treated with ETV because of the worry about NUCs resistance.

11 patients (9.9%) had been treated with NUCs previously for long-terms (range from 43 to 312 weeks, median: 104 weeks) and subsequently interrupted or discontinued the NUCs treatments till the occurrence of AD events. The interruption periods were range from 9 to 267 weeks, with a median as 26 weeks. 8 of them were previously treated with ETV, one with LAM and 2 with adefovir dipivoxil. ETV treatment was started after the day of admission in 10 of them. One patient was treated with TDF due to a confirmed ETV resistance mutation.

Table 1 indicated that previous NUCs treatment could effectively inhibit HBV replications and interruption/discontinuation of NUCs treatment might lead to viral relapse, but it did not influence the incidence of ACLF development (*P* > 0.05) .

### Association between serum HBV DNA ad-level and incidence of ACLF or short-term mortality rate (Table 2, Table 3)

Serum HBV DNA ad-levels did not significantly vary between the 43 cases who developed ACLF and the 68 cases who did not (all *P*>0.05). Respective analyses based on the data of the 26 cases diagnosed with ACLF at admission and 17 cases developed ACLF after admission got consistent conclusion with the previous analysis (all *P*>0.05) (Table 2). In the 39 ACLF cases with detailed 28-day medical records and without LT treatment, serum HBV DNA ad-levels of the 6 patients died in 28 days and the 33 patients survived till the 28th days were similar (logarithms: 4.31 ± 1.91 vs 5.54 ± 2.53, *P*>0.05). In the 22 ACLF cases with detailed 90-day medical records and without LT treatment, serum HBV DNA ad-levels of the 9 patients died in 90 days were not significantly different from that of those survived till the 90th days (logarithms: 4.81 ± 1.76 vs 4.84 ± 2.40, *P*>0.05). The short-term LT-free mortality rates of ACLF patients with serum HBV DNA ad-levels lower than 2000 IU/ml or 2 × 10^6^ IU/ml did not vary from those of higher (both *P*>0.05) (Table 3).

**Table 2:** Serum HBV DNA ad-levels of the patients who developed to ACLF or not

Groups	n	HBV DNA (IU/ml), n (%)						Log (HBV DNA), mean ± SD
		<10	≥10	<2000	≥2000	<2 × 10^6^	≥2 × 10^6^	
Non-ACLF	68	4 (5.9)	64 (94.1)	22 (32.4)	46 (67.6)	56(82.4)	12(17.6)	4.32 ± 1.99
ACLF before admission	26	1 (3.80)	25 (96.2)	7 (26.9)	19 (73.1)	20(76.9)	6(23.1)	4.65 ± 1.99
ACLF after admission	17	0 (0.0)	17 (100.0)	7(41.2)	10 (58.8)	15(88.2)	2(11.8)	4.27 ± 1.94
Total	111	5 (4.5)	106 (95.5)	36 (32.4)	75 (67.6)	91(82.0)	20(18.0)	4.39 ± 1.97

1. Groups: Non-ACLF, patients without ACLF development; ACLF, patients with ACLF development;

2. HBV DNA <10 IU/ml or ≥ 10 IU/ml: Fisher’s exact test, *P* = 0.827

3. HBV DNA <2000 IU/ml or ≥ 2000 IU/ml: χ^2^ = 0.953, *P* = 0.597

4. HBV DNA <2 × 10^6^ IU/ml or ≥ 2 × 10^6^ IU/ml: Fisher’s exact test, *P* = 0.643

5. Log(HBV DNA) (logarithm of the serum HBV DNA ad-level): F = 0.304, *P* = 0.739

**Table 3:** The 28-day or 90-day LT-free mortality rates in the ACLF patients with serum HBV DNA ad-level lower or higher than 2000 IU/ml or 2 × 10^6^ IU/ml

HBV DNA (IU/ml)	28-day prognosis		90-day prognosis	
	n	mortality, n(%)	n	mortality, n(%)
<2000	13	1 (7.7)	7	3 (42.9)
≥2000	26	5 (19.2)	15	6 (40.0)
<2 × 10^6^	31	4 (12.9)	17	7 (41.2)
≥2 × 10^6^	8	2 (25.0)	5	2 (40.0)
Total	39	6 (15.4)	22	9 (40.9)

1. LT: liver transplantation

2. HBV DNA <2000 IU/ml or ≥ 2000 IU/ml: 28-day LT-free mortality rates: Fisher’s exact test, *P* = 0.643; 90-day LT-free mortality rates: Fisher’s exact test, *P* = 1.000

3. HBV DNA <2 × 10^6^ IU/ml or ≥ 2 × 10^6^ IU/ml: 28-day LT-free mortality rates: Fisher’s exact test, *P* = 0.583; 90-day LT-free mortality rates: Fisher’s exact test, *P* = 1.000

### Logistic analysis of the 28- or 90-day mortality rates in patients without LT treatment

We performed a binary logistic analysis of the 28- or 90-day mortality rates in patients without LT treatment. The 28 or 90 days outcomes of the patients with or without ACLF development were set as dependent variables. Gender, age, ALT, MELD score and logarithm of the serum HBV DNA ad-level were set as covariates. 106 cases were involved in the 28-day analysis, and 55 cases were included in the 90-day analysis. The results showed that MELD score at admission, i.e. the degree of liver decompensation, was the main factor affecting the recent mortality of the patients (OR = 1.139, OR95% CI:1.006–1.289, *P* = 0.040; OR = 1.259, OR95% CI:1.049–1.512, *P* = 0.014, respectively). The other factors, including HBV DNA ad-level, had not significant influences (all *P* > 0.05).

## Discussion

According to the EASL-CLIF diagnostic criteria, the CLIF-SOFA scores and the prevalence of ACLF in the 111 patients enrolled were consistent with the reports from Shanghai Municipality and Zhejiang Province, China [3, 5]. Comparison of MELD scores at admission between the patients developing ACLF and those not, indicated that proper treatment scheme in the early stage of AD was vital to reduce ACLF incidence. However, there was no statistical correlation between previous NUCs treatment and ACLF incidence. It meant that, although continuous NUCs therapy could reduce the risk of AD event among cirrhotic patients [6–9], once AD occurred, the previous NUCs treatment would not interfere the ACLF development.

This assumption was also consistent with the analysis of PEs. Similar to other reports [1, 3–5], interruption/discontinuation of NUCs therapies was one of the frequent PEs associated with AD or ACLF in the 111 patients. HBV replication level was significantly higher in the patients with interruption/discontinuation of antiviral treatments, but there was no difference in the proportion of ACLF development among the antiviral treatment-naïve patients and the NUCs treatment-experienced patients with or without interruptions. According to the reports from Shanghai, China, patients with ACLF had been more frequently treated with NUCs within the 6 months prior to admission than patients with AD without ACLF [3]. It was interesting that ACLF incidence and short-term mortality rate of the patients without presence of PEs were also not related to the HBV DNA ad-level.

Obviously, ACLF incidence and short-term mortality rate were mainly dependent on the severity of systemic inflammation and the number of organ failures, not on the levels of HBV replication of the patients. Many evidences suggested that development of ACLF was associated with many factors such as abnormal immune reaction, imbalance of pro-inflammatory and anti-inflammatory cytokines, and so on [29, 30]. So the urgent treatments in the hospitalized acutely decompensated CHB-related cirrhotic patients were reducing the inflammatory responses and maintaining the major organs such as liver and kidney so as to reduce ACLF development and decrease mortality rate.

However, NUCs treatment was still necessary for the hospitalized CHB-related cirrhotic patients [12–15]. The incidence of ACLF and short-term mortality rate in the acutely decompensated cirrhotic patients were different from the incidence of AD and HCC and long-term mortality rate in the cirrhotic patients. Effective anti-viral therapies would reduce the risk of HCC and decompensated liver disease among the cirrhotic patients [6–9]. Regression of fibrosis and even reversal of liver cirrhosis have been reported in patients with prolonged suppression of HBV replication [31]. Besides, NUCs treatments were well tolerated in acutely decompensated patients, without significant side effects [9, 10]. As shown in the current study, the previous NUCs treatments of the 23 patients did not increase the incidence of ACLF or the short-term mortality rate. So, continuous NUCs treatments from the admission days were recommended in view of their long-term prognoses.

Although the cases recruited were limited and the dynamic changes of the HBV DNA levels around the occurrences of AD events were inavailable, the conclusion of the current study could still be credible. It was consistent with other data, although it was not the emphases of the other reports [1, 3–5]. So, for the hospitalized patients with AD of CHB-related cirrhosis, besides NUCs treatment, physicians should pay more attention to other approaches to reduce ACLF incidence.

## Abbreviations

ACLF: acute-on-chronic liver failure
AD: acute decompensation
CHB: chronic hepatitis B
CI: Confidence Intervals
CTP score: Child-Turcotte-Pugh (CTP) score
DNA: deoxyribonucleic acid
HBV DNA ad-level: serum HBV DNA levels at admission
HBV: hepatitis B virus
HCC: hepatocellular carcinoma
MELD score: a model for end-stage liver disease score
NUCs: nucleoside/nucleotide analogues
OR: odds ratio
PaO2: partial pressure of arterial oxygen
PCR: polymerase chain reaction
PE: precipitating event
PTA: prothrombin activity
SD: standard deviation
SpO2: pulse oximetric saturation
